# An Asymmetrical Hypothesis for the NREM-REM Sleep Alternation—What Is the NREM-REM Cycle?

**DOI:** 10.3389/fnins.2021.627193

**Published:** 2021-04-08

**Authors:** Olivier Le Bon

**Affiliations:** Laboratory of Psychiatric Research (ULB 266), Department of Psychiatry, Cliniques Universitaires de Bruxelles, Université libre de Bruxelles (ULB), Bruxelles, Belgium

**Keywords:** NREM (Non-REM) sleep, rem, sleep, regulation, model, alternation, cycle, hypothesis

## Abstract

Since the discovery of rapid eye movement (REM) sleep (Aserinsky and Kleitman, 1953), sleep has been described as a succession of cycles of non-REM (NREM) and REM sleep episodes. The hypothesis of short-term REM sleep homeostasis, which is currently the basis of most credible theories on sleep regulation, is built upon a positive correlation between the duration of a REM sleep episode and the duration of the interval until the next REM sleep episode (inter-REM interval): the duration of REM sleep would therefore predict the duration of this interval. However, the high variability of inter-REM intervals, especially in polyphasic sleep, argues against a simple oscillator model. A new “asymmetrical” hypothesis is presented here, where REM sleep episodes only determine the duration of a proportional post-REM refractory period (PRRP), during which REM sleep is forbidden and the only remaining options are isolated NREM episodes or waking. After the PRRP, all three options are available again (NREM, REM, and Wake). I will explain why I think this hypothesis also calls into question the notion of NREM-REM sleep cycles.

## Introduction

Since the discovery of rapid eye movement sleep (REM) by Aserinsky and Kleitman (1953), we know that sleep is composed of two distinct neurophysiological states: rapid eye movement (REM) and non-rapid eye movement (NREM). Despite intensive research, the functional relationship between REM and NREM remains a matter of great conjecture.

Several theories are competing to explain this relationship within what is commonly referred to as a “NREM-REM sleep cycle,” or “ultradian sleep cycle.” Humans have between 2 and 8 such cycles during the course of a night and the most common pattern is 4–5 cycles of about 90 min (see Le Bon et al., 2019, for more on this distribution). Rodents may exhibit 100–250 cycles per 24 h. In a recent article, I discussed these theories and presented their respective advantages and disadvantages (Le Bon, 2020).

## Rationale

### Correlations Between REM and Immediately Consecutive Intervals vs. NREM Sleep Content

With the exception of the Energy Allocation theory, which focuses on resource optimization (Schmidt, 2014), all credible hypotheses on the NREM-REM cycle are currently based on a correlation that was established separately by two research teams in 1994 (Benington and Heller, 1994; Vivaldi et al., 1994). The duration of a REM sleep episode is related to the duration of the immediately consecutive inter-REM interval (Vivaldi et al., 1994), and its NREM content (Benington and Heller, 1994). These findings in rats have been confirmed in other mammals: cats (Ursin, 1970), monkeys (Weitzman et al., 1965; Kripke et al., 1968), mice (Le Bon et al., 2007; Weber et al., 2018), and humans (Barbato and Wehr, 1998).

However, some studies have also found correlations between REM sleep episodes and previous NREM episodes, or a mixture of the two, in rats (Vivaldi et al., 1994; Franken, 2002), cats (Ursin, 1970), and humans (Hartmann, 1966; Barbato and Wehr, 1998).

Comparisons between the duration of REM sleep episodes and the spectral power of NREM episodes have also produced contrasting results. In humans, no clear general trend was observed with either previous or consecutive episodes (Le Bon and Linkowski, 2013), while a positive correlation was found in rodents between REM sleep duration and the spectral power of subsequent NREM episodes (Hayashi et al., 2015).

The main correlation (between REM sleep and the consecutive inter-REM interval) was thus confirmed by the majority of reports on multiphasic sleepers, but the balance is more uneven in monophasic humans. From this point on, “inter-REM interval” will always refer to immediately consecutive REM sleep intervals.

After interventions combining total sleep deprivation and REM sleep deprivation (Ocampo-Garcés et al., 2000), and total sleep deprivation (Franken, 2002), we have understood that although a short-term NREM sleep-dependent regulation of REM sleep cannot be ruled out (NREM Renewal theories, see Benington and Heller, 1994), REM sleep pressure accumulates mainly in a long-term process.

What is most important in the correlation between the duration of a REM sleep episode and the inter-REM interval is essentially the duration of the inter-REM interval itself, not the relative proportions of its NREM and/or Wake content. This interval thus seems to be primarily a matter of REM sleep, by REM sleep and for REM sleep.

### Short-Term and Long-Term REM Sleep Homeostasis

The hypothesis deduced from this correlation between REM and the immediately consecutive inter-REM interval is called “Short-term REM sleep homeostasis.” First put forward by Vivaldi et al. (1994), it was reformulated by Franken (2002). The Short-term REM sleep homeostasis would determine the timing of the new REM sleep episode. Cycles comprising the REM sleep episode and the inter-REM interval would be formed, including any NREM or Wakefulness episodes that may occur between them. In the Short-term REM sleep homeostasis, the REM sleep pressure would wax and wane, cycles would reflect a slow brain rhythm (Weber, 2017), we could speak of cycles, ultradian rhythms and oscillators. Franken (2002) also added the notion of a Long-term REM sleep homeostasis, which would be responsible for the intensity of REM pressure, which we will discuss later.

**FIGURE 1:**
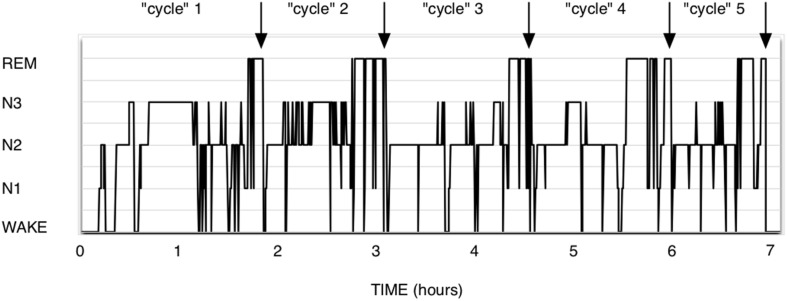
Human polysomnogram. Healthy male, 35 years. It is intuitive here to think in terms of cycles, since about the same NREM duration precedes the 5 presented REM sleep episodes. It could be interesting to exercise the eye to see them differently, as PRRP with a null Lambda and immediate return to NREM sleep.

**FIGURE 2:**
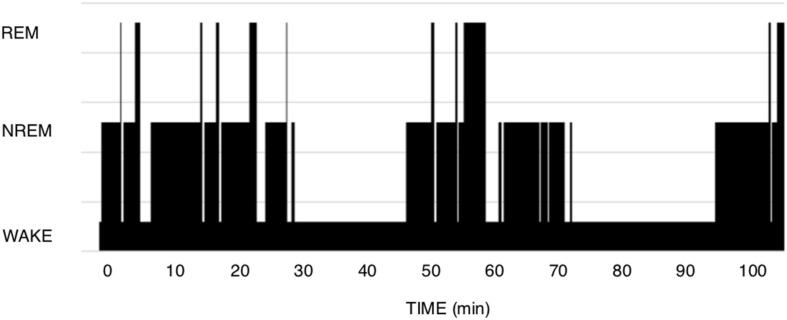
Mouse polysomnogram. CD1 mouse, 7 months old. We observe here a large variety of inter-REM intervals as well as an intercurrent mix of long Wake episodes, irregular sequences, isolated NREM episodes. In comparison to human hypnograms, it is more difficult here to detect the positive relationship between the duration of a REM episode and the inter-REM duration.

The theory of Short-term homeostasis poses some problems. (1) Although the shape of polysomnograms of young healthy humans often suggests the existence of NREM-REM sleep cycles, it requires more imagination and some computing when examining recordings of polyphasic sleepers such as rodents. In human polysomnograms, nights often show the expected fairly regular alternation, as in Figure 1, but can also sometimes show much more irregular patterns, which are difficult to explain by this theory. In rodent polysomnograms (Figure 2), the duration of inter-REM intervals is highly variable: relatively long periods without alternation or isolated NREM episodes are commonly observed—among other things because the entire waking life of the animal takes place between two REM episodes (leaving the two extremes of life open). Paradoxically, the correlation between REM and the inter-REM interval is stronger in polyphasic sleepers than in monophasic sleepers, as discussed above. This is probably related to the much greater number of REM episodes in rodents than in humans over 24 h.

(2) The mechanism by which the REM pressure prompts a new REM episode is unknown and could be complex. In a true cycle, a decreasing and increasing REM pressure should at some point “call” for a new REM episode. Since REM sleep never starts a sequence in adults, this can only be done by interrupting an NREM episode, so we should imagine a compound mechanism where REM sleep first calls for a new NREM episode. Then, after a given time in NREM sleep, REM sleep would inhibit it.

Clear oscillations can be observed in most hormones depending on the REM or NREM status during sleep. Growth hormone, prolactin, plasma renin, plasma catecholamines, insulin, and glucagon increase during NREM sleep and decrease abruptly during REM sleep (see Born and Fehm, 2000, for example). Thus, the levels of these hormones correspond to the sleep state. What we lack, in my opinion, to support the expected resurgence of REM sleep episodes, is a neurosubstrate (or any other alternative mechanism) that would anticipate the return of REM sleep and close the cycle. Otherwise, the resurgence of REM is more likely to be the result of the convergence of internal and/or external semi-random circumstances, as in the hypothesis that I will develop below.

(3) In a study in mice (Weber et al., 2018), the GABAergic Ventro-lateral periaqueductal gray (VlPAG) REM-off firing was found to be maximal after the end of REM sleep episodes and then reduced at each consecutive isolated NREM sleep episode—but not during intermittent Wake episodes. The vlPAG GABA firing level therefore decreases gradually until a new REM episode occurs, at which point the vlPAG firing stops completely. Although the authors did not specify it, I understand this as an argument against the Short-term REM sleep homeostasis. In this theory, as we have seen above, it is the inter-REM intervals (the combined durations of the NREM and Wake episodes) that are the dependent variable of previous independent REM sleep episode durations. Rather, the absence of vlPAG reduction during Intermittent Wake tends to support, in my opinion, a more binomial relationship between REM and NREM, such as that proposed in the Renewal theories described above.

### Some Basic Principles of the Alternation Between the Two Sleep States

Before I can present my alternative “Asymmetrical” REM sleep hypothesis, we need to review some fundamental elements of the regulation of sleep alternation.

Although they are still only virtual concepts, it is generally considered that a NREM pressure determines the behavior of NREM sleep, and a REM pressure determines the behavior of REM sleep. Both pressures would reflect circadian, homeostatic and allostatic influences, as well as the effects of the respective selective sleep debts and whatever the sleep states’ (still largely uncovered) functions are specifically for. The respective moments of NREM and REM pressures may therefore vary from night to night in humans, or in 24 h in rodents.

The degree of dependence between NREM and REM is a fundamental issue. From a semi-historical perspective, there has been a trend toward greater independence between sleep states, from the early and very rigid Basic Rest-Activity cycle (Kleitman, 1961), to the Renewal-type (Benington and Heller, 1994), to the Limit-Cycle Reciprocal Interaction Model (McCarley and Hobson, 1975; McCarley and Massaquoi, 1986), to the Short-term REM sleep homeostasis (Vivaldi et al., 1994). The Asymmetrical hypothesis is the one that currently proposes the greatest independence between the states.

Since some NREM is always necessary for a REM sleep episode to occur in adults, REM sleep cannot be completely independent of NREM. But evidence also supports the existence of separate regulations for sleep states. For example: (1) Total NREM sleep deprivation was followed by NREM sleep restoration (Borbély et al., 1981). Deprivations of stages 1, 2 and REM were also followed by NREM restoration (Brunner et al., 1993). REM sleep deprivation was followed by restoration of REM sleep (Endo et al., 1998; Le Bon et al., 2001); (3) REM/NREM ratios over one night (monophasic humans) or 24 h (polyphasic rodents) were found to be more correlated with total REM sleep than with total NREM sleep, so that more cycles are preferentially related to more total REM sleep (Le Bon et al., 2002, 2007, 2019). These ratios also vary with age. In altricial species such as humans, infants spend 80–90% of their time sleeping, and about half this sleep time is spent in REM sleep. This represents 40–70% of REM sleep in 24 h. The ratio between REM and NREM sleep is there much higher than in adults.

The alternation always begins with a NREM sleep episode. After a delay that is a function of the respective NREM and REM pressures, REM sleep first inhibits and then interrupts NREM. If the NREM pressure is very high (as at the beginning of human nights), attempts by the REM pressure to create a REM episode may fail (see skipped REM episodes further). If the NREM pressure is less high, a REM episode appears but may be terminated by the NREM pressure before what could be its potentially maximal duration. If the NREM pressure is lower, “full-fledged” REM episodes may appear, that never last more than a given maximal duration (see more on this later). After such full-fledged REM episodes, the animal either returns to NREM or wakes up.

Isolated NREM episodes, not interrupted by REM sleep, account for 25–75% of total NREM sleep in mice (Le Bon et al., 2007; Weber et al., 2018). Since these isolated NREM episodes may be at varying distances from the nearest REM episode, deciding what is a “pre-REM” NREM, “post-REM” NREM or “isolated” NREM is a matter of definition and tolerance to awakening (Le Bon et al., 2007). There is no consensus on these issues.

### Post-REM Refractory Period

A Post-REM Refractory Period (PRRP) has been proposed independently in two recent papers (Le Bon, 2020; Ocampo-Garces et al., 2020). It would determine a duration during which a new episode of REM sleep is unlikely to occur. This PRRP could therefore only be used for Wake or NREM sleep.

The firing of the GABAergic VlPAG (REM-off) cells at the end of a REM sleep episode, followed by its progressive decay described in the Weber et al. (2018) paper cited above, may well reflect the PRRP proposed here (see section “Discussion”).

### The Asymmetrical Hypothesis on NREM-REM Alternation

Since there are two options at the end of each REM sleep episode (NREM and Wake), it is unlikely that REM sleep determines what follows it immediately. It also makes sense for the body that thermally-unstable REM sleep does not last too long. In the present hypothesis, if REM sleep episodes attain their potentially maximal duration (full-fledged REM sleep episodes), they limit their own duration. A negative feedback loop of some kind must be initiated at REM sleep onset to obtain that result. This loop is short-circuited if the REM episode is terminated by NREM or an awakening before having reached this potentially maximal duration.

At the end of a REM sleep episode, the REM pressure would be inhibited by maximal REM-off firing, which thus corresponds to the beginning of the PRRP. During this PRRP, only two options remain: NREM or Wake. The duration of the PRRP would be proportional to the duration of the REM sleep episode, its inhibition of new REM sleep episodes would be progressively lower. The probability of a new REM sleep episode would therefore be relative: very unlikely at the beginning of the PRRP—increasingly likely toward its end. Being purely theoretical at this stage, it is impossible to determine by what factor the REM sleep episode would have to be multiplied to obtain the PRRP, but it is very likely to be greater than one.

The NREM pressure will sooner or later impose new NREM sleep episodes, for reasons *proper to itself and to Wake*, unrelated to the REM pressure.

**FIGURE 3:**
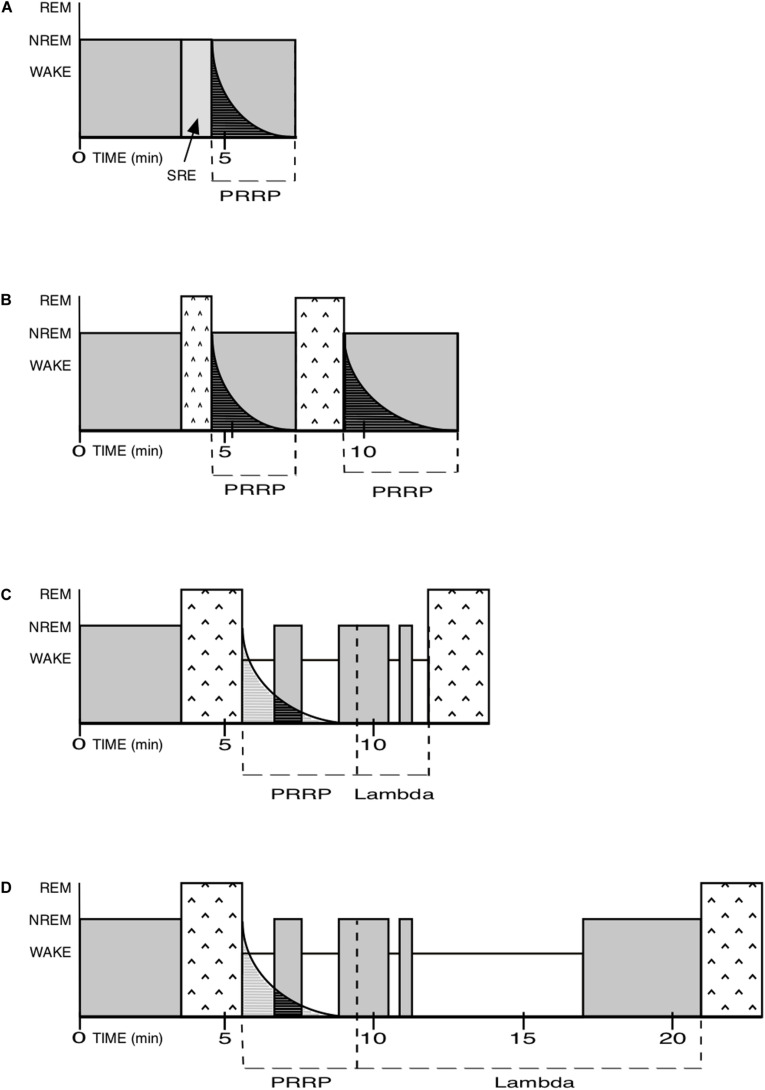
NREM and REM pressure, PRRP and Lambda. PRRP for Post-REM refractory period. SRE for Skipped REM Episode. In order to simplify the examples below, the REM pressure is assumed to be constant here, whereas in reality it is determined by its long-term pressure, and may also be higher or lower. In real nights, it is the balance between both pressures that will determine the local patterns. Please note that the time scale used here is for mice and should be adapted for other species. **(A)** As in human nights, especially at their beginning, SRE: the NREM pressure is very high and completely blocks the attempt by the REM pressure to create a REM episode. (SRE are only evidenced by a lighter level of NREM sleep, see p.7). The PRRP then in turn also blocks attempts by the REM pressure to create a new REM sleep episode. **(B)** As in human nights, especially at their beginning: the NREM pressure is high but loses some ground. It prevents the REM pressure to create a “full-fledged” first REM episode and forces it to shut down after a while. The PRRP following it blocks any attempt by the REM pressure to create a new REM episode. When the PRRP is complete, a new, usually longer, episode of REM sleep is created, followed by a proportionally longer PRRP than the first one. The Lambda duration is zero in in the first case and indeterminate in the second. **(C)** Human (mostly at the end of nights) or rodent: the NREM pressure is lower here and allows a full-fledged REM episode to appear. Full-fledged REM sleep episodes limit their own duration. The PRRP then prevents REM sleep pressure to launch a new REM episode too rapidly after the one just ended. The Lambda duration is relatively short in the example. **(D)** Human (for example in cases of insomnia) or rodent: same as above but with a longer Lambda.

Between the PRRP and the onset of the next REM sleep episode, there is a territory of indeterminate duration, which includes a mix of isolated NREM episodes and Wake time. I call it Lambda. The sum of PRRP and Lambda is equivalent to the inter-REM interval (Figure 3).

Lambda could be zero, when a new REM episode immediately follows the PRRP, it can be shorter or longer. It would be long, for example, when the next REM sleep episode occurs after a prolonged period of Wake corresponding to activities such as foraging, flight from predators, reproduction, rest, etc.

This division of the inter-REM interval into PRRP and Lambda has the advantage of allowing fairly variable distances between REM episodes, as observed in the field, whereas the Short-Term REM sleep homeostasis pleads for an oscillator and more regular durations within cycles.

The REM pressure would be “ambushed,” waiting for a propitious circumstance. A REM sleep episode would be created when three conditions are met: (a) a sufficiently high level of REM pressure; (b) a sufficiently large NREM episode; (c) no inhibition by the PRRP.

With each episode of REM sleep, the REM sleep pressure would be reduced accordingly. Its curve between two episodes of REM sleep would reflect its long-term buildup. The question of whether it increases exclusively during Wake time, during NREM sleep episodes, or both, has not been clearly answered so far (see section “Discussion”).

Higher REM pressure would result in more frequent detour of NREM episodes to REM sleep—thus there would be fewer isolated NREM episodes overall. REM episodes could also be longer (see Franken, 2002; Hayashi et al., 2015).

The maximum duration of REM sleep episodes and PRRP would both be species-specific. Indeed, links have been found between brain size (Zepelin, 2000) and body mass (Elgar et al., 1990). Small animals with small brain size tend to have more frequent and smaller episodes of REM sleep than large animals. This is probably related to the surface/volume ratio of the animals and the lack of thermoregulation during REM sleep (see Schmidt, 2014).

If the current hypothesis is correct, the “NREM-REM cycle” is an optical illusion, especially in monophasic sleep. It would represent an epiphenomenon of PRRP and other contributing factors, such as the Long-term pressure.

The NREM-REM cycle would be replaced by a triad NREM-REM-PRRP, followed by the Lambda described above. The triad would constitute compounds against the background of Wake and isolated NREM episodes. NREM and REM would alternate asymmetrically, always in the same, but not necessarily complete, NREM-REM-PRRP-Lambda sequence.

### “Skipped” REM Episodes

As seen above, cases where REM sleep does not seem strong enough to interrupt NREM sleep are well known to observers of human nights as “skipped,” or “aborted” cycles (Dement and Kleitman, 1957). Here, NREM (Slow Wave Sleep, one of its components, to be more precise) shows a rapid decrease, so that an episode of REM sleep episode is “expected” to appear—except that it does not. Then NREM increases again. A PRRP would also be present in these cases, because, to my knowledge, there is no description in the literature of a REM episode immediately following a skipped REM.

## Discussion

### A New Hypothesis on the NREM-REM Sleep Alternation

On the basis of the problems related to Short-term homeostasis and the recent hypothesis of a PRRP, an Asymmetrical hypothesis is made that the duration of an REM sleep episode only determines the duration of a PRRP, and not the timing of the next REM episode. Such an asymmetric hypothesis would be more flexible than existing assumptions.

To summarize the Asymmetrical hypothesis: (1) NREM would not have an ultradian cyclicity (it does have a circadian cyclicity, however). Left to itself—would there be no REM sleep—it would wax and wane according to its relationship with Wake and the awakened life of the animal. Its regulation would be very flexible. (2) The organism, for a reason that is still largely obscure, also needs REM sleep. To appear, REM sleeps requires a minimal duration and/or power of immediately preceding NREM sleep. REM sleep would take control by inhibiting and interrupting NREM sleep (probably REM-on cells inhibiting the firing of REM-off cells). This may fail (skipped REM episodes) or succeed for a period of time. If the NREM pressure is high, such as at the beginning of human nights, the REM episode will be terminated by the NREM pressure and the episode will not attain its (species-specific) potentially maximal duration. If the NREM pressure is lower, as at the end of human nights, the REM episode may not be obstructed and attain its maximal duration (“full-fledged” REM sleep episodes). (3) To prevent the duration of these (full-fledged) REM episodes from being too long and putting the animal at behavioral or thermal risk (see Schmidt, 2014), REM sleep is limited by some mechanism or neurohormonal negative feedback loop (REM-off cells). When REM episodes are terminated by the high NREM pressure, or by awakenings, the self-limitation mechanisms that I am proposing here is short-circuited. (4) A PRRP is generated in all cases: when REM is not expressed at all (skipped REM episodes), when REM sleep episodes are interrupted by NREM or Wake or when the REM episode is not interrupted (full-fledged). (5) The PRRP would progressively degrade and be increasingly compatible with a return to REM sleep. During the PRRP, only the two remaining states can appear: NREM sleep or Wake. The duration of the REM sleep episode and the PRRP are both species-specific. (6) After the progressive decay of the PRRP, the system returns to a situation where REM sleep is again possible. The lapse of time between the end of the PRRP and the next episode of REM sleep is called Lambda. (7) The REM pressure will sooner or later divert a compatible NREM episode when it finds one, to create a new REM sleep episode, and so on.

The Asymmetrical hypothesis would be compatible with most polysomnograms, for both monophasic and polyphasic species. It could even be compatible with bird sleep, for which no cyclicity is claimed. There would be no need for an ultradian REM sleep oscillator and no slow brain rhythm would be suggested. I see no contradictions between this hypothesis and REM flip-flop (Lu et al., 2006; Weber, 2017).

The present hypothesis is also compatible with the Energy Allocation (Schmidt, 2014) theories. For example, Komagata et al. (2019) showed in a study on mice that melanin-concentrating hormone (MCH) neurons in the hypothalamus play a key role in the dynamic increase of REM sleep expression with an increase in REM sleep episodes during thermoneutral warming. This shows that REM sleep can be expressed opportunistically under ideal conditions and be reduced instead in environments that are too cold or too warm. This also represents an argument against fixed lengths of inter-REM intervals.

### Correlations Between a REM Episode Duration and the Inter-REM Interval

The repeatedly confirmed correlation between the duration of REM sleep episodes and the duration of the inter-REM interval, on which the hypothesis of the Short-term REM sleep homeostasis is based, is in apparent contradiction with the Asymmetrical hypothesis. A similar problem is the correlation found the study by Weber et al. (2018) between these variables and the progressive decay in VlPAG.

The large variance of the inter-REM interval in polyphasic sleepers is one of the main reasons why the Asymmetrical hypothesis was borne. We can assume that these correlations are due, on one hand, to the temporal compression method used to standardize the duration of REM sleep episode the inter-REM interval and the VlPAG decay, and, on the other hand, to the averaging of inter-REM intervals by time units. These strategies are useful for creating variables that can be compared statistically, but there is a risk is of exceeding their conclusions.

By averaging the inter-REM intervals, it is impossible to distinguish between PRRP and Lambda. In a few samples, the correlation is probably low. But in a large number of sequences, Lambda would have no reason to escape a Gaussian distribution, and the duration of the interval (PRRP + Lambda) would finally be correlated with the previous REM sleep duration, but only indirectly. It may be useful to compare these variables on an episode-by-episode basis to avoid averaging.

### During Which Stages Does the REM Pressure Increase?

A major question is whether the REM pressure increases during NREM episodes, Wake episodes, or both. Two reference articles (Ocampo-Garcés et al., 2000) and Franken (2002) have shown that REM pressure at least partially increases during the time spent out of NREM sleep. The intensity of the REM pressure is thought to be due to a Long-term homeostasis process. This logically implies time spent in Wake.

On the other hand, Weber et al. (2018) suggested that VlPAG GABAergic firing reflects REM pressure. Here, the decay of VlPAG (i.e., in this case, the recharge of REM pressure), would happen only during isolated NREM episodes, not during the Wake time. (Note that in the Asymmetrical hypothesis, VlPAG firing would reflect the PRRP instead of the REM pressure).

What then happens with the long-term REM sleep homeostasis? In the first case, REM would be needed because of Wake time activities. In the second, it would be to balance NREM sleep in a more binomial relationship.

We do not have enough elements today to reconcile these results and the question remains open. Other mechanisms may be at play.

### The Asymmetrical Hypothesis and the Long-Term REM Sleep Homeostasis

The present hypothesis does not challenge the Long-term REM sleep hypothesis (Franken, 2002), but its implementation is somewhat different than if we use the Short-term REM sleep hypothesis.

As proposed above, a REM episode would be created when three conditions are met: (a) a sufficiently high level of REM pressure; (b) a sufficiently large NREM episode; (c) no inhibition by PRRP. A higher REM pressure would result in more frequent deviations of NREM episodes for REM sleep purposes—thus there would be fewer isolated NREM episodes overall and REM episodes could be longer (see Franken, 2002; Hayashi et al., 2015).

The duration of a REM episode here determines only the duration of the PRRP, not the duration of the inter-REM interval. Lambda, on the other hand, is influenced by the (long term) REM pressure. Thus, the higher the REM pressure, the higher the REM frequency, the longer the REM sleep episodes and the fewer the isolated NREM episodes. When the REM pressure is low, more and/or longer isolated NREM episodes may occur.

### The Issue of NREM and REM Sleep “Cycling” in Mammals

I have not been able to find the first occurrence of the term “cycle” or “cycling” referring to NREM and REM sleep, but, to my knowledge, there have never been any alternative proposals either. The present (uncontested) Wikipedia definition of the sleep cycle is “an oscillation between slow-wave and REM (paradoxical) phases of sleep.” This definition may suggest that the sleep tissue consists of harmonious and symmetrical ultradian switching from one type of sleep to another. It may be time to question this consensus.

There are obvious behavioral differences between mammalian monophasic and polyphasic sleep patterns. It is, however, difficult to imagine that they respond to different basic rules, all the more so since many species, felines for example, present a mix of the two. Monophasic adult humans can also take one or more naps during the day, infants and the elderly do so frequently. There are also cultural and adaptive reasons for taking more naps when the sun is scorching and preventing human activity, or for waking up in the middle of the night when the sun sets at 4 p.m. (Ekirch, 2005).

I would therefore tend to consider monophasic sleep as an extreme case of sleep compaction and deepening, allowing minimal periods of wakefulness during the sleep period, and maximum periods of wakefulness outside of it. If we accept this continuum between monophasic and polyphasic sleep types, we need to find an alternation mechanism that accommodates both. Although monophasic sleep is what we know more closely and surely are most interested in, it may not be the best material to work on if we want to understand the transitions from wakefulness to NREM and REM sleep.

In the monophasic sleep of a healthy adult (Figure 1), the notion of NREM-REM cycles is intuitive. The pattern is roughly similar from person to person and from night to night: NREM sleep begins as a rule, then alternates more or less regularly with REM sleep, and the sleep ends with either a NREM phase or a REM sleep phase. Sleep is deeper at the beginning of the night and REM sleep is more frequent in the second tier. There are between 2 and 8 REM episodes per night, which, depending on the definition of sleep cycles, means that we have 2–8 “cycles.”

In polyphasic sleep, we are more clearly dealing with the three metabolic states in which our brain can function. It is much more difficult here to describe typical cycles (Figure 2). Polyphasic sleepers such as rodents present sleep as taking place in episodes of various sequences: NREM-REM-Wake; isolated NREM between two episodes of Wake; NREM-REM-NREM or NREM-Wake, among other more complex. Since NREM-REM “cycles” are also defined by the presence of a single REM sleep episode at their end, these cycles include varying proportions of NREM and Wake, and certainly do not have an immutable order or regular periodicity. As we have seen above, the cycle reported by species is the result of an arithmetic division (number of REM sleep episodes per 12 or 24 h) more than the mirror of what is observed “cycle by cycle” on a time scale. A mouse can thus present 100–250 REM episodes, and thus, depending on the definition, 100–250 “cycles.” Note that if we counted NREM episodes instead of REM episodes, we could multiply this frequency by a factor of 2–4.

No matter how irregular this repeated appearance of REM may be, it will always be possible to average the results in units of time and summarize this phenomenon as the product of cycles. These averages allow the use of statistical comparisons, such as those that correlate the cycle with brain or body size, or the relationship between a REM sleep episode and the inter-REM interval.

But if what is observed has ultimately no regular timing, no fixed sequence and variable REM/NREM ratios, it might be more accurate to speak of average frequency and average duration of sleep episodes than cycles. These will be just as species-specific as the current cycles.

For all these reasons, I consider that there is no real cycling between NREM and REM sleep. Both sleep states follow their own logic. They are forced to alternate but without much harmony or regularity.

Therefore, either the term “cycling” is taken very broadly, or we should start looking for a more adequate, though perhaps less intuitive, expression to describe the sequence of events that occur between the three states.

## Conclusion

In the Short-term homeostasis theory, a REM sleep episode can approximately predict the timing of the next REM sleep episode. An underlying mechanism must be at work for the next episode of REM sleep to occur sooner or later. Cycling, even loose and irregular, eventually presupposes some form of regularity.

In the Asymmetrical hypothesis, the prediction after a REM sleep episode is limited to the duration of the PRRP. No underlying mechanism is presupposed, there is no ultradian oscillator and no slow brain rhythm. This allows for more varied patterns from one REM episode to another. Since the maximum limit of REM and PRRP duration is species-specific, shorter animals will have more cycles than larger ones.

In order to demonstrate the value of the Asymmetrical hypothesis, important steps will be necessary: (1) measure the mathematical relationship between the duration of the REM and its PRRP; (2) confirm the parallel between the firing GABAergic VlPAG and the PRRP, or find other biological substrates to explain it; (3) Model the Assymetrical hypothesis and compare it with the Short-Term homeostasis to verify which one fits better with reality.

## Data Availability Statement

The original contributions presented in the study are included in the article/supplementary material, further inquiries can be directed to the corresponding author.

## Author Contributions

OL: conception, editing, and drawing.

## Conflict of Interest

The author declares that the research was conducted in the absence of any commercial or financial relationships that could be construed as a potential conflict of interest.

## References

[B1] Aserinsky E. Kleitman N. (1953). Regularly occurring periods of eye motility, and concomitant phenomena, during sleep. *Science* 118 273–274. 10.1126/science.118.3062.273 13089671

[B2] Barbato G. Wehr T. A. (1998). Homeostatic regulation of REM sleep in humans during extended sleep. *Sleep* 21 267–276 10.1093/sleep/21.3.267 9595605

[B3] Benington J. H. Heller H. C. (1994). REM-sleep timing is controlled homeostatically by accumulation of REM-sleep propensity in non-REM sleep. *Am. J. Physiol.* 266 R1992–R2000 8024056 10.1152/ajpregu.1994.266.6.R1992

[B4] Borbély A. A. Baumann F. Brandeis D. Strauch I. Lehmann D. (1981). Sleep deprivation: effect on sleep stages and EEG power density in man. *Electroencephalogr. Clin. Neurophysiol.* 51 483–495 10.1016/0013-4694(81)90225-x 6165548

[B5] Born J. Fehm H. L. (2000). The neuroendocrine function of sleep. *Noise Health* 2 25–38 12689469

[B6] Brunner D. P. Dijk D. J. Borbély A. A. (1993). Repeated partial sleep deprivation progressively changes in EEG during sleep and wakefulness. *Sleep* 16 100–113 10.1093/sleep/16.2.100 8446828

[B7] Dement W. Kleitman N. (1957). Cyclic variations in EEG during sleep and their relation to eye movement, body motility, and dreaming. *Electroencephalogr. Clin. Neurophysiol.* 9 673–690 10.1016/0013-4694(57)90088-3 13480240

[B8] Ekirch A. R. (2005). *At Day’s Close – Night in Times Past.* New York, NY: WW Norton & Company

[B9] Elgar M. A. Pagel M. D. Harvey H. (1990). *Sources of Variation in Mammalian Sleep. Anim. Behav.* 5:991e5

[B10] Endo T. Roth C. Landolt H. P. Werth E. Aeschbach D. Achermann P. Borbély A. A. (1998). Selective REM sleep deprivation in humans: effects on sleep and sleep EEG. *Am. J. Physiol.* 274 R1186–R1194 9575987 10.1152/ajpregu.1998.274.4.R1186

[B11] Franken P. (2002). Long-term vs. short-term processes regulating REM sleep. *J. Sleep Res.* 11 17–28 10.1046/j.1365-2869.2002.00275.x 11869422

[B12] Hartmann E. (1966). Mechanism underlying the sleep-dream cycle. *Nature* 212 648–650 10.1038/212648b0 5971699

[B13] Hayashi Y. Kashiwagi M. Yasuda K. Ando R. Kanuka M. Sakai K. Itohara S. (2015). Cells of a common developmental origin regulate REM/non-REM sleep and wakefulness in mice. *Science* 350 957–961 10.1126/science.aad1023 26494173

[B14] Kleitman N. (1961). The nature of dreaming. in *The Nature of Sleep*, eds Wolstenholme GEW O’Connor M (London: Churchill)

[B15] Komagata N. Latifi B. Rusterholz T. Bassetti C. L. A. Adamantidis A. Schmidt M. H. (2019). Dynamic REM sleep modulation by ambient temperature and the critical role of the melanin-concentrating hormone system. *Curr. Biol.* 29 1976–1987. 10.1016/j.cub.2019.05.009 31155350

[B16] Kripke D. F. Crowley T. J. Pegram G. V. Halberg F. (1968). Circadian rhythmic amplitude modulation of Berger-region frequencies in electroencephalograms from *Macaca mulatta*. *Rass Neurol. Veg.* 22 519–525 4988188

[B17] Le Bon O. Lanquart J. P. Hein M. Loas G. (2019). Sleep ultradian cycling: statistical distribution and links with other sleep variables, depression, insomnia and sleepiness - a retrospective study on 2,312 polysomnograms. *Psychiat. Res.* 279 140–147 10.1016/j.psychres.2018.12.141 30819535

[B18] Le Bon O. Linkowski P. (2013). Absence of systematic relationships between REMS duration episodes and spectral power Delta and Ultra-Slow bands in contiguous NREMS episodes in healthy humans. *J. Neurophysiol.* 110 162–169 10.1152/jn.00020.2013 23596336

[B19] Le Bon O. Popa D. Streel E. Alexandre C. Lena C. Linkowski P. Adrien J. (2007). Ultradian cycles in mice: definitions and links with REMS and NREMS. *J. Comp. Physiol. A Neuroethol. Sens. Neural. Behav. Physiol.* 193 1021–1032 10.1007/s00359-007-0253-7 17724599

[B20] Le Bon O. Staner L. Hoffmann G. Dramaix M. San Sebastian I. Murphy J. R. Kentos M. Pelc I. Linkowski P. (2001). The first-night effect may last more than one night. *J. Psychiatr. Res.* 35 165–172 10.1016/s0022-3956(01)00019-x 11461712

[B21] Le Bon O. Staner L. Rivelli S. K. Hoffmann G. Pelc I. Linkowski P. (2002). Correlations using the NREM-REM sleep cycle frequency support distinct regulation mechanisms for REM and NREM sleep. *J. Appl. Physiol.* 93 141–146 10.1152/japplphysiol.00917.2001 12070197

[B22] Le Bon O. (2020). Relationships between REM and NREM in the NREM-REM sleep cycle: a review on competing concepts. *Sleep Med.* 70 6–16 10.1016/j.sleep.2020.02.004 32179430

[B23] Lu J. Sherman D. Devor M. Saper C. B. (2006). A putative flip-flop switch for control of REM sleep. *Nature* 441 589–594 10.1038/nature04767 16688184

[B24] McCarley R. W. Hobson J. A. (1975). Neuronal excitability modulation over the sleep cycle: a structural and mathematical model. *Science* 189 58–60. 10.1126/science.1135627 1135627

[B25] McCarley R. W. Massaquoi S. G. (1986). A limit cycle mathematical model of the REM sleep oscillator system. *Am. J. Physiol.* 251 R1011–R1029 3789188 10.1152/ajpregu.1986.251.6.R1011

[B26] Ocampo-Garces A. Bassi A. Brunetti E. Estrada J. Vivaldi E. (2020). REM sleep-dependent short-term and long-term hourglass processes in the ultradian organization and recovery of REM sleep in the rat. *Sleep* 12:43 10.1093/sleep/zsaa023 32052056

[B27] Ocampo-Garcés A. Molina E. RodriÆguez A. Vivaldi E. A. (2000). Homeostasis of REM sleep after total and selective sleep deprivation in the rat. *J. Neurophysiol.* 84 2699–2702 10.1152/jn.2000.84.5.2699 11068012

[B28] Schmidt M. H. (2014). The energy allocation function of sleep: a unifying theory of sleep, torpor, and continuous wakefulness. *Neurosci. Biobehav. Rev.* 47 122–153 10.1016/j.neubiorev.2014.08.001 25117535

[B29] Ursin R. (1970). Sleep stage relations within the sleep cycles of the cat. *Brain Res.* 20 91–97 10.1016/0006-8993(70)90157-5 4315521

[B30] Vivaldi E. A. Ocampo A. Wyneken U. Roncagliolo M. Zapata A. M. (1994). Short-term homeostasis of active sleep and the architecture of sleep in the rat. *J. Neurophysiol.* 72 1745–1755 10.1152/jn.1994.72.4.1745 7823099

[B31] Weber F. Hoang Do J. P. Chung S. Beier K. T. Bikov M. Saffari Doost M. Dan Y. (2018). Regulation of REM and Non-REM Sleep by Periaqueductal GABAergic Neurons. *Nat. Commun.* 9:354 10.1038/s41467-017-02765-w PMC5783937 29367602

[B32] Weber F. (2017). Modeling the mammalian sleep cycle. *Curr. Opin. Neurobiol.* 46 68–75 10.1016/j.conb.2017.07.009 28841438

[B33] Weitzman E. D. Kripke D. F. Pollak C. Dominguez J. (1965). Cyclic activity in sleep of *macaca mulatta*. *Arch. Neurol.* 12 463–467 10.1001/archneur.1965.00460290019003 14288983

[B34] Zepelin H. (2000). “Mammalian sleep,” in *Principles and Practice of Sleep Medicine.* 3rd ed, eds Kryger M. H. Roth T. Dement W. C. (Philadelphia, PA: Saunders)

